# COVID-19-related music-video-watching among the Vietnamese population: lessons on health education

**DOI:** 10.3934/publichealth.2021033

**Published:** 2021-05-18

**Authors:** Nguyen Tuan Hung, Vu Thu Trang, Trinh Van Tung, Nguyen Xuan Long, Ha Thi Thu, Tran Song Giang, Tran Hoang Thi Diem Ngoc, Vu Thi Thanh Mai, Nguyen Kim Oanh, Nguyen Thi Phuong, Nguyen Hang Nguyet Van, Nguyen Hanh Dung, Pham Tien Nam

**Affiliations:** 1 Ministry of Health, Hanoi, Vietnam; 2 Graduate Academy of Social Sciences, Hanoi, Vietnam; 3 University of Social Sciences and Humanities, Vietnam National University, Hanoi, Vietnam; 4 University of Languages and International Studies, Vietnam National University, Hanoi, Vietnam; 5 Bach Mai Hospital, Hanoi, Vietnam; 6 Thanglong University, Hanoi, Vietnam; 7 Hanoi University of Public Health, Hanoi, Vietnam

**Keywords:** health education, music-video-watching, COVID-19, Vietnamese population, lessons

## Abstract

**Background:**

Health education through music video plays a vital role in raising a person's knowledge, attitudes, and behaviors positively connected to health during COVID-19 pandemic.

**Objective:**

This study aimed to estimate the prevalence of COVID-19-related music-video-watching and examine associated factors among the Vietnamese population.

**Methods:**

A cross-sectional study in Vietnam was conducted in February 2021 via the Internet.

**Results:**

Among 658 participants, the prevalence of COVID-19-related music-video-watching was 89.1% among people. In the multivariable regression models, significant factors for COVID-19-related music-video-watching were living area, types of housemate, age groups, and current occupation.

**Conclusions:**

Lessons on health education to fight against the COVID-19 pandemic in Vietnam could be useful for similar settings in the world.

## 1. Introduction

The COVID-19 pandemic is a global health crisis and threatening human lives, especially vulnerable groups such as people living in poverty situations, older persons, persons with disabilities, youth, and indigenous peoples [1]. According to the report on 16 February 2021, the total number of people diagnosed with COVID-19 was over 108,2 million cases, with over 2,3 million deaths globally since the start of the pandemic [2]; among these, 2,311 cases have been reported in Vietnam [3]. The prevalence of confirmed COVID-19 cases in Vietnam was lower than in some countries in Southeast Asia such as Thailand, Philippines, Malaysia, and developed countries such as China, South Korea, Japan, USA, UK, etc. [2]. Vietnam has contained the COVID-19 pandemic with limited resources due to the direction of the Communist Party and the Government of Vietnam and comprehensive community health actions [4]. Among these actions, health education campaigns have been carried out through music videos to raise community awareness about COVID-19 and promote hygienic practices such as using a face mask, washing hands with soap, etc. [4]. The Integrated Theory of Health Behavior Change suggests that health behavior change can be enhanced by fostering knowledge and beliefs, increasing self-regulation skills and abilities, and enhancing social facilitation. Music-video-watching has a great influence in changing people's perceptions and health behaviors [5].

In Vietnam, there have been a few studies on the COVID-19 pandemic that focus on clinical aspect [6], mental health problems and quality of life among people with suspected COVID-19 symptoms [7],[8], and the Vietnam lessons of policy response, social media, and science jouralism for the COVID-19 outbreak [9]. There have been no studies on COVID-19-related music-video-watching among people. In this study, we aimed to estimate the prevalence of COVID-19-related music-video-watching among the Vietnamese population and to examine the association between various factors which are not well reported in previous studies. We hypothesized that the sociodemographic characteristics of participants such as age groups, gender, marital status, types of housemate, geographical regions, living area, current occupation, and self-reported anxiety may affect certainly COVID-19-related music-video-watching among the Vietnamese population during COVID-19 pandemic.

## 2. Materials and methods

### 2.1. Study design and setting

We conducted a cross-sectional study on people from three geographical regions in Vietnam (Northern, Central, and Southern Vietnam). The survey was carried out in February 2021.

### 2.2. Study participant

People were recruited based on the following criteria: (1) being at least 12 years old; (2) living in Vietnam during a survey period; (3) agreeing to enroll in the study; (4) having the ability to answer the google form via the Internet.

### 2.3. Study sample size and sampling

This study would be using the formula of a population proportion to calculate the sample size.

$$n=Z_{1–}^{2}\alpha_{/2}\cdot [p\cdot x\cdot (1–p)]/d^{2}$$ (1)

in which: n = sample size; p = 0.5 (currently there are no studies on the COVID-19-related music-video-watching among the Vietnamese population, so p = 0.5 to get the maximum sample size); absolute precision required (d) = 0.05; Z1–α/2 is the value from the standard normal distribution, calculated based on statistical significance (Z1–α/2 = 1.96 if statistical significance = 0.05)

According to the above sample size formula, n = 385 people. In this study, a self-nomination sampling technique was applied to recruit participants. Any person could access and complete the google form if he/she wanted to participate in our survey according to eligibility criteria. Therefore, the final sample size of this study included 658 respondents.

All potential people were explained about the study's purpose, and informed consent was then obtained to confirm their enrollment.

### 2.4. Study variables

Dependent variable: COVID-19-related music-video-watching was measured as watching a completed music video within the past month. The answers were then categorized to (1) yes, I watched it, and (2) No, I did not watch it.

If people answered (1) yes, I watched it, they would specify the name of popular music videos watched within the past month: (1) “Ghen Cô Vy” (Jealous Coronavirus), (2) “Việt Nam ơi! Đánh bay Covid” (Vietnam! Fighting against Covid), (3) “Việt Nam sẽ chiến Thắng” (Vietnam will win), (4) “Sao anh chưa về nhà” (Why haven't you come home yet?), (5) “Bao la những trái tim hồng” (Immensely pink hearts), and (6) “Chống giặc Corona như chống giặc ngoại xâm” (Fighting against Covid-19 like Fighting against invaders). Information channels of knowing COVID-19-related music video were measured by four indicators: (1) television, (2) social media, (3) relatives' sharing, and (4) friends' sharing. Furthermore, the importance of COVID-19-related music video elements was categorized as: (1) lyrics, (2) melody, (3) anti-epidemic images, (4) the appearance of famous singers, and (5) choreography. The effect of COVID-19-related music-video-watching was grouped as follows: (1) washing hands with soap, (2) using a face mask, (3) raising awareness of physical activity, (4) feeling more comfortable, (5) raising awareness of social distancing, (6) feeling proud and grateful to the frontline doctors, (7) raising awareness of non-discrimination and non-stigma against people infected with COVID-19, and isolated people, and (8) raising awareness of fake news on COVID-19.

Independent variables: Sociodemographic variables were included, such as (1) age groups (12–18 years old, 19–35 years old, 36–55 years old, and over 55 years old), (2) gender (male and female), (3) marital status (single, married, and other), (4) types of housemate (living alone, and living with family or roommate), (5) geographical regions (Northern Vietnam, Central Vietnam, and Southern Vietnam), (6) living area (rural and urban areas), (7) current occupation (junior high school students and high school students, university students, civil servants, freelance labor, retirement, and housewife), and (8) self-reported anxiety (yes and no).

### 2.5. Study instruments

The questionnaire included questions about the sociodemographic characteristics (age groups, gender, marital status, types of housemate, geographical regions, living area, current occupation, and self-reported anxiety), COVID-19-related music-video-watching (name of COVID-19-related music videos watched within the past month, information channels of knowing COVID-19-related music videos, importance of COVID-19-related music video elements, effect of COVID-19-related music-video-watching).

To screen for anxiety symptoms among people during the outbreak of Covid-19, we used the Generalised Anxiety Disorder Questionnaire (GAD-7). The optimal balance between sensitivity and specificity for the GAD-diagnosis was found to be a cut-off point of ≥10 [10]–[12]. Therefore, we categorized participants scoring 10 points and above as screened positive for anxiety symptoms.

### 2.6. Data analysis

We used Chi-square by Stata 14.2 Survey package to compare the differences in sociodemographic factors. A multivariate Poisson regression model with robust error variances was performed to examine associated factors for COVID-19-related music-video-watching [13]–[15]. We calculated Prevalence Ratios (PRs), together with corresponding 95% Confidence Interval (CI), and used a significance level of p < 0.05.

### 2.7. Ethical considerations

The protocol of this study was approved by the Institutional Review Board, Vietnam Association of Psychology. Participants' information was completely confidential and only served for study purposes. In this study, participants' involvement was voluntary without any incentives.

## 3. Results

### 3.1. General characteristics of the study sample

Table 1 illustrates the sociodemographic characteristics of respondents. In a total of 658 people, 89.1% of people (95% CI: 0.86–0.91) watched COVID-19-related music videos within the past month. The majority of respondents in our survey were female (72.3%), aged 19–35 years old (62.6%), single (65.8%), and living with family or roommates (92.6%). Regarding living area, the survey respondents reported coming from three geographical regions in Vietnam (Northern, Central, and Southern Vietnam). Among these regions, more than half of respondents were coming from Northern Vietnam (67.3%). The prevalence of people living in urban areas was 55.9%. The respondents' current occupation is very diverse, including junior high school students and high school students, university students, civil servants, freelance labor, retirement, and housewife. Among these occupations, university students accounted for the highest prevalence (41.8%). We found that the prevalence of living alone, living in rural areas, and having anxiety symptoms were higher in those who do not watch music videos compared to those who watch music videos.

**Table 1.** Sociodemographic characteristics of respondents.

Columns by: COVID-19-related music-video-watching	No	Yes	Total	P-value
n (%)	72 (10.9%)	586 (89.1%)	658 (100.0)	
Age groups, n (%)				**<0.001**
12–18 years old, n (%)	3 (4.2%)	79 (13.5%)	82 (12.5%)	
19–35 years old, n (%)	25 (34.7%)	387 (66.0%)	412 (62.6%)	
36–55 years old, n (%)	21 (29.2%)	116 (19.8%)	137 (20.8%)	
Over 55 years old, n (%)	23 (31.9%)	4 (0.7%)	27 (4.1%)	
Gender, n (%)				0.76
Male, n (%)	21 (29.2%)	161 (27.5%)	182 (27.7%)	
Female, n (%)	51 (70.8%)	425 (72.5%)	476 (72.3%)	
Marital status, n (%)				**0.001**
Single, n (%)	44 (61.1%)	389 (66.4%)	433 (65.8%)	
Married, n (%)	23 (31.9%)	191 (32.6%)	214 (32.5%)	
Other, n (%)	5 (6.9%)	6 (1.0%)	11 (1.7%)	
Types of housemate, n (%)				**<0.001**
Living alone, n (%)	18 (25.0%)	31 (5.3%)	49 (7.4%)	
Living with family or roommate, n (%)	54 (75.0%)	555 (94.7%)	609 (92.6%)	
Geographical regions, n (%)				0.73
Northern Vietnam, n (%)	48 (66.7%)	395 (67.4%)	443 (67.3%)	
Central Vietnam, n (%)	7 (9.7%)	71 (12.1%)	78 (11.9%)	
Southern Vietnam, n (%)	17 (23.6%)	120 (20.5%)	137 (20.8%)	
Living area, n (%)				**<0.001**
Urban areas, n (%)	20 (27.8%)	348 (59.4%)	368 (55.9%)	
Rural areas, n (%)	52 (72.2%)	238 (40.6%)	290 (44.1%)	
Current occupation, n (%)				**<0.001**
Junior high school students and high school students, n (%)	5 (6.9%)	75 (12.8%)	80 (12.2%)	
University students, n (%)	22 (30.6%)	253 (43.2%)	275 (41.8%)	
Civil servants, n (%)	10 (13.9%)	176 (30.0%)	186 (28.3%)	
Freelancer, n (%)	29 (40.3%)	76 (13.0%)	105 (16.0%)	
Retirement and housewife, n (%)	6 (8.3%)	6 (1.0%)	12 (1.8%)	
Self-reported anxiety, n (%)				**<0.001**
No, n (%)	52 (72.2%)	570 (97.3%)	622 (94.53%)	
Yes, n (%)	20 (27.8%)	16 (2.7%)	36 (5.47%)	

Note: Statistical comparison using: Chi-square test for categorical variable—display as n (%);The bold p-value indicated statistical significance (p < 0.05).

### 3.2. COVID-19-related music-video-watching

COVID-19-related music-video-watching of respondents is demonstrated in Table 2. Around 94.7% of people watched the music video “Ghen Cô Vy” (Jealous Coronavirus). COVID-19-related music videos were transmitted through information channels such as television, social media, and sharing of relatives or friends, in which social media accounted for the highest prevalence (97.3%). People reported the importance of COVID-19-related music video elements such as lyrics (98.0%), anti-epidemic images (93.7%), and melody (92.2%). Most people who watched COVID-19-related music videos recognized the positive effects on the prevention of COVID-19 pandemics, such as: using a face mask (98.3%), raising awareness of physical activity (95.4%), washing hands with soap (95.2%), feeling proud, and grateful to the frontline doctors (95.1%), raising awareness of social distancing (94.5%), feeling more comfortable (93.9%), raising awareness of not sharing fake news on COVID-19 (92.8%), and raising awareness of non-discrimination and non-stigma against people infected with COVID-19, and isolated people (88.7%).

**Table 2.** COVID-19-related music-video-watching among the Vietnamese population within the past month.

	Yes	No	Total
Name of COVID-19-related music videos watched within the past month			586 (100%)
“Ghen Cô Vy” (Jealous Coronavirus)	555 (94.7%)	31 (5.3%)	
“Việt Nam ơi! Đánh bay Covid” (Vietnam! Fighting against Covid)	439 (74.9%)	147 (25.1%)	
“Sao anh chưa về nhà” (Why haven't you come home yet?)	302 (51.5%)	284 (48.5%)	
“Việt nam sẽ chiến thắng” (Vietnam will win)	421 (71.8%)	165 (28.2%)	
“Bao la những trái tim hồng” (Immensely pink hearts)	151 (25.8%)	435 (74.2%)	
“Chống giặc Corona như chống giặc ngoại xâm” (Fighting against Covid-19 like Fighting against invaders)	231 (39.4%)	355 (60.6%)	
Information channels of knowing COVID-19-related music videos			586 (100%)
Television	477 (81.4%)	109 (18.6%)	
Social media	570 (97.3%)	16 (2.7%)	
Relatives' sharing	353 (60.2%)	233 (39.8%)	
Friends' sharing	412 (70.3%)	174 (29.7%)	
Importance of COVID-19-related music video elements			586 (100%)
Lyrics	574 (98.0%)	12 (2.0%)	
Melody	540 (92.2%)	46 (7.8%)	
Anti-epidemic images	549 (93.7%)	37 (6.3%)	
Appearance of famous singers	271 (46.2%)	315 (53.8%)	
Choreography	400 (68.3%)	186 (31.7%)	
Effect of COVID-19-related music-video-watching			586 (100%)
Washing hands with soap	558 (95.2%)	28 (4.8%)	
Using a face mask	576 (98.3%)	10 (1.7%)	
Raising awareness of physical activity	559 (95.4%)	27 (4.6%)	
Feeling more comfortable	550 (93.9%)	36 (6.1%)	
Raising awareness of social distancing	554 (94.5%)	32 (5.5%)	
Feeling proud and grateful to the frontline doctors	557 (95.1%)	29 (4.9%)	
Raising awareness of non-discrimination and non-stigma against people infected with COVID-19, and isolated people.	520 (88.7%)	66 (11.3%)	
Raising awareness of fake news on COVID-19	544 (92.8%)	42 (7.2%)	

### 3.3. Associated factors with COVID-19-related music-video-watching

**Table 3.** The multivariable regression model.

	Model		
	PR	95% CI	
Age groups			
12–18 years old	REF		
19–35 years old	0.74	0.53–1.03	
36–55 years old	0.64	0.45–0.90	**
Over 55 years old	0.13	0.05–0.35	***
Gender			
Male	REF	.	
Female	0.98	0.93–1.03	
Marital status			
Single	REF	.	
Married	1.02	0.93–1.10	
Other	1.05	0.87–1.28	
Types of housemate			
Living alone	REF	.	
Living with family or roommate	1.18	1.02–1.40	*
Living area			
Urban areas	REF	.	
Rural areas	0.89	0.84–0.94	***
Current occupation			
Junior high school students and high school students	REF	.	
University students	1.32	0.93–1.86	
Civil servants	1.41	0.99–2.00	
Freelancer	1.23	0.87–1.75	
Retirement and housewife	1.72	1.05–2.81	*
Self-reported anxiety			
No	REF	.	
Yes	0.88	0.71–1.08	
N = 658			

Note: * p < 0.05, ** p < 0.01, *** p < 0.001.

The results of the multivariable regression model are presented in Table 3. The association between COVID-19-related music-video-watching and factors such as living area, types of housemate, age groups, and current occupation were statistically significant in our study. Regarding the age groups, the prevalence of COVID-19-related music-video-watching among people aged 36–55 years old was only 0.64 compared to people aged 12–18 years old (PR = 0.64, 95% CI: 0.45–0.90). This prevalence was 87.0% lower among people aged over 55 years old (PR = 0.13, 95% CI: 0.05–0.35). The prevalence of COVID-19-related music-video-watching in rural areas was 11.0% lower than that of urban areas (PR = 0.89, 95% CI: 0.84–0.94). Living with family or roommates also increased the prevalence of COVID-19-related music-video-watching by a factor of 1.20 among people (PR = 1.20, 95% CI: 1.02–1.40). Retirement and housewife had a higher prevalence of COVID-19-related music-video-watching (PR = 1.72, 95% CI: 1.05–2.81) compared to the junior high school students and high school students.

## 4. Discussion

Our result shows that the prevalence of COVID-19-related music-video-watching among Vietnamese people was quite high. Besides, we tested the initial hypothesis. The association between living area, types of housemate, age groups, and current occupation and COVID-19-related music-video-watching were statistically significant.

Our survey time coincided with the mandatory period of social distancing in some parts of the country [16]. Therefore, people could spend time watching COVID-19-related music videos for entertainment and access information on preventing the COVID-19 pandemic.

Among the music videos, “Ghen Co Vy” (Jealous Coronavirus), which was based on the melody of the V-pop hit “Ghen” was written by Khac Hung, in collaboration with the National Institute of Occupational and Environmental Health under the Ministry of Health. This COVID-19-related music video was well-known worldwide and highlighted on the latest episode of HBO's Last Week Tonight With John Oliver and the French channel [17]. The lyrics of “Ghen Co Vy” (Jealous Coronavirus) call on viewers to wash their hands thoroughly, not touch their faces, avoid large crowds, and “push back the virus corona, corona” [18].

In our study, the information channel of knowing COVID-19-related music videos was mostly via social media, including Facebook, Zalo, etc. In Vietnam, 57.34% of the population reported facebook usage [19], while there were around 100 million Zalo users [20]. Vietnam government, especially the Ministry of Health, has provided citizens with COVID-19-related information in a timely way via social media [9].

Previous studies pointed out that music videos significantly influence the awareness and behavior of viewers [21]–[23]. Our results show that COVID-19-related music videos may increase awareness and behavior among Vietnamese people amid the COVID-19 outbreak, such as face mask usage, hand-washing, sanitation, the rising awareness in physical activity, social distancing, etc. These are basic protective measures against the COVID-19 outbreak according to the advice of the World Health Organization and Vietnam's Ministry of Health [24],[25].

In the regression models, we demonstrated the relationship between COVID-19-related music-video-watching and living area. The prevalence of COVID-19-related music-video-watching among people living in rural areas was lower than those living in urban areas. This could be explained by the fact that people living in rural areas spend less time watching music videos via television and social media than people living in urban areas [26]. Moreover, according to Vietnamese rural culture, people often have an early dinner and then go to bed.

Data from this study also shows an association between COVID-19-related music-video-watching and types of housemate. In Vietnam, the relationship among family members and close friends are quite strong [27], and they could watch together COVID-19-related music video at home during social distancing. Therefore, the prevalence of COVID-19-related music-video-watching among people living with family or roommate was higher than those living alone.

We found evidence of a significant association between COVID-19-related music-video-watching and age groups. The prevalence of COVID-19-related music-video-watching among people aged 36–55 years old and over 55 years old was lower than that of other age groups in our study. There may be an explanation that people aged 36–55 years old could be busy at work; therefore, they have less time to watch COVID-19-related music videos. People aged over 55 years old could use less social media [28] to watch COVID-19-related music videos compared to other age groups. Furthermore, COVID-19-related music videos may not match the style of this age group. World Health Organization emphasized that older people are at the highest risk from COVID-19, and they need to have protective measures to prevent the further community spread of the virus [29].

In the regression models, we also demonstrated that current occupation showed an association with COVID-19-related music-video-watching. The prevalence of COVID-19-related music-video-watching among retirement and housewife was higher than that of junior high school students and high school students. This result might be due to retirement and housewife have more free time to watch COVID-19-related music videos than junior high school students and high school students.

There are two limitations to our study. Firstly, causal relationship could not be determined from this cross-sectional study. Secondly, one of the limitations of the study is its representativeness. This is because the internet access and collection varies from region to region.

## 5. Conclusions and lessons on health education

The prevalence of COVID-19-related music-video-watching among Vietnamese people was relatively high. This watching led to health education's positive effect to prevent the COVID-19 outbreak in Vietnam. We demonstrated the relationship between COVID-19-related music-video-watching and various factors such as living area, types of housemate, age groups, and current occupation. We hope that prospective studies will confirm our findings. Furthermore, some lessons on health education to fight against the COVID-19 pandemic in Vietnam could be useful for similar settings in the world, such as: promoting health education activities through COVID-19-related music video to improve people's knowledge, attitudes, and behaviors in the prevention of the COVID-19 pandemic; paying attention to associated factors for COVID-19-related music-video-watching such as living area, types of housemate, age groups, and current occupation; enhancing communication activities to introduce COVID-19-related music video via social media, youTube or broadcast live on news programs; creating anti-epidemic songs based on the rewrite of old hit; using the famous singers to sing the songs; incorporating the images of anti-epidemic health workers into video clips; creating dance challenges via social media or making clip contests.
